# The Synthesis of Blood Group Antigenic A Trisaccharide and Its Biotinylated Derivative

**DOI:** 10.3390/molecules26195887

**Published:** 2021-09-28

**Authors:** Ekaterina D. Kazakova, Dmitry V. Yashunsky, Nikolay E. Nifantiev

**Affiliations:** Laboratory of Glycoconjugate Chemistry, N. D. Zelinsky Institute of Organic Chemistry, Russian Academy of Sciences, Leninsky pr. 47, 119991 Moscow, Russia; edkazakova@gmail.com (E.D.K.); yashunsky1959@yandex.ru (D.V.Y.)

**Keywords:** blood group determinants, carbohydrates, stereoselective glycosylation

## Abstract

Blood group antigenic A trisaccharide represents the terminal residue of all A blood group antigens and plays a key role in blood cell recognition and blood group compatibility. Herein, we describe the synthesis of the spacered A trisaccharide by means of an assembly scheme that employs in its most complex step the recently proposed glycosyl donor of the 2-azido-2-deoxy-selenogalactoside type, bearing stereocontrolling 3-O-benzoyl and 4,6-O-(di-tert-butylsilylene)-protecting groups. Its application provided efficient and stereoselective formation of the required α-glycosylation product, which was then deprotected and subjected to spacer biotinylation to give both target products, which are in demand for biochemical studies.

## 1. Introduction

Since the discovery of the ABO blood group system and the role of carbohydrate residues in blood antigens [1,2], there has been continued interest in developing new synthetic approaches to the assembly of carbohydrate blood group antigen determinants [3,4,5,6,7,8,9,10]. Besides playing an important role in blood cell recognition and blood group compatibility, blood transfusion, and organ transplantation [11,12,13], A trisaccharide and structurally related compounds can be used as haptens to test the carbohydrate specificities of plant [14] and mammalian lectins [15,16] and serve as a model for conformational and spectral studies [17] of vicinally branched oligosaccharides. A trisaccharide derivatives can also serve as model compounds in the development of new biomedical technologies, since antibodies against this carbohydrate antigen are commercially available.

**Figure 1:**
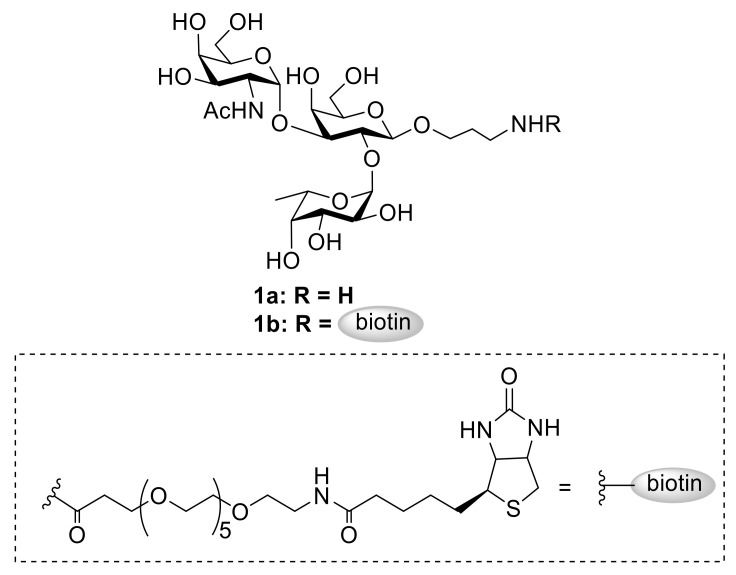
Target spacered A trisaccharide derivatives **1a** and **1b**.

A trisaccharide represents the minimal terminal fragment of all blood group A antigens. It has a branched structure where the central β-Gal residue is glycosylated with α-fucose at O-2 and with α-galactosamine at O-3 (see Figure 1). Despite numerous works devoted to the synthesis of oligosaccharides related to blood group antigens, there are only a few papers dedicated specifically to the synthesis of A trisaccharide derivatives [4,5,6,9]. Herein, we report on the assembly of spacered A trisaccharide **1a** and its biotinylated derivative **1b**, making use of the new galactose block **4**, which bears a set of convenient temporary protecting groups permitting selective liberation of HO-groups as well fucosyl donor **5** [18,19] and bicyclic 2-azido-2-deoxy-selenogalactoside **10** [20] containing stereo-controlling O-protecting groups, which favor the required α-(1,2-*cis*)-glycosylation. A bulky 4,6-O-(di-tert-butylsilylene)-protecting group at O-4 and O-6 was used to prevent the formation of undesirable β-glycosylation products [21] while a 3-O-benzoyl group was introduced to provide α-stereocontrol through remote anchimeric participation [22].

Donor **10** was recently proposed [20,23] and has been successfully applied in the syntheses of complex linear oligosaccharides related to bacterial and fungal antigenic polysaccharides (see a mini-review [23] and references therein). At the same time, there is still little published data on the glycosylation of vicinally branched oligosaccharides by means of 2-azido-2-deoxy-selenogalactoside donors as well as the application of the donors in the regioselective glycosylation of diolic acceptors. The studies discussed below were planned to fill these gaps.

## 2. Results and Discussion

**Scheme 1:**
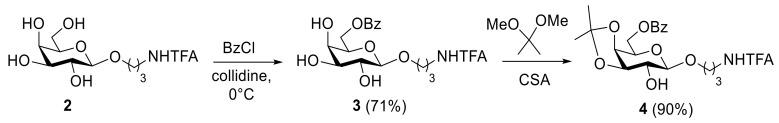
Synthesis of galactosyl acceptor **4**.

Key steps in the synthesis of spacered A trisaccharide **1a** were the regio- and stereoselective building of three glycosidic bonds. While the formation of a β-glycoside bond is a straightforward task, α-glycosylation by fucosyl and galactosamine donors requires the careful selection of protective groups and experimental conditions effective for α-glycoside bond formation [24]. To promote the desired stereoselectivity, a new type of galactosyl acceptor **4** was easily synthesized from tetraol **2** [25] through the introduction of 3,4-O-isopropylidene and 6-O-benzoyl groups, which can be selectively removed in the presence of other protecting O-substituents (Scheme 1).

**Scheme 2:**
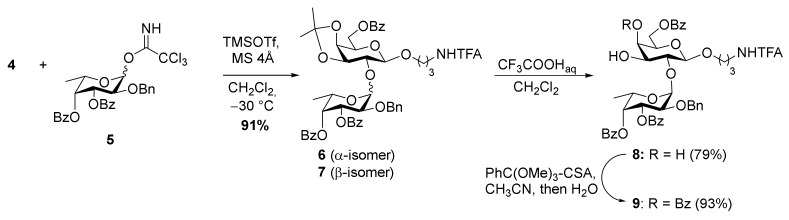
Synthesis of disaccharides **8** and **9**.

To form the fucosyl block, donor **5** was used. This compound contains two benzoyl protecting groups at O-3 and O-4, which, despite reducing the donor’s activity, provide effective α-directing glycosylation stereocontrol through remote anchimeric participation [18,19]. Fucosylation of galactoside **4** proceeded stereoselectively, giving an inseparable mixture of α-isomer **6** and βisomer **7** (Scheme 2) in the ratio ~9:1 with a yield of 91%. The anomeric configurations of the Fuc units in disaccharides **6** and **7** were confirmed by the characteristic values of the corresponding C-1 signals in ^13^C NMR spectra and *J*_1,2_ constants in the 1H NMR spectra (for **6**: 95.5 ppm and 3.4 Hz; for **7**: and 103.3 ppm and 8.0 Hz, respectively). The individual α-isomer was purified after the removal of the O-isopropylidene group, which gave the desired diol **8** in a 79% yield. The value of coupling constant *J*_1,2_ (3.6 Hz) confirmed the α-configuration of the Fuc unit in **8**. In addition to diol **8,** its monohydroxy-derivative **9** was prepared by treatment with trimethyl orthobenzoate [26] followed by hydrolytic opening of the 3,4-O-orthobenzoic intermediate in an overall yield of 93% (Scheme 2). The presence of the 4-O-benzoyl group was confirmed by a downfield shift of the H-4Gal signal by 1.77 ppm between the spectra for compounds **8** and **9**.

The last step in the assembly of the trisaccharide A backbone was the glycosylation of disaccharide **8** with 2-azido-2-deoxy-galactosyl donor **10**, which was prepared via azidophenylselenylation of a triacetylgalactal [23,27] and subsequent selective protection. The glycosylation α-stereoselectivity of donors of this type can be regulated by the reaction solvent [28] and specially selected types of O-protective groups [20,21,22,23,29,30].

**Scheme 3:**
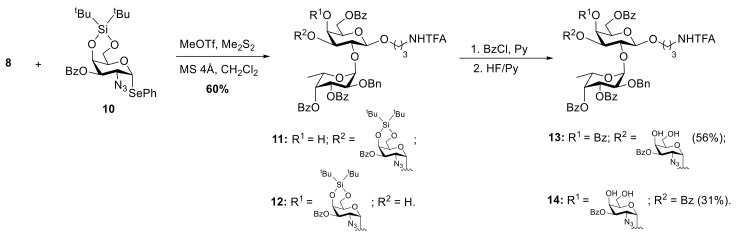
Glycosylation of disaccharide **8** with donor **10**.

It is known that equatorial hydroxyl groups are usually more reactive than axial ones [31,32]. Based on this assumption, we suggest that a regioselective 3-O-glycosylation of diol **8** would be possible. However, the reaction between disaccharide **8** and donor **10** yielded an inseparable mixture of products (Scheme 3). Presumably, it consisted of regioisomers **11** and **12** in a 2.8:1 ratio (NMR data), which were formed via (1→3)- and (1→4)-glycosylation, respectively.

To check our assumption, we treated the glycosylation products with BzCl in Py and then removed the 4,6-O-(di-tert-butylsilylene)-protection with HF/Py. As result, we obtained two separate compounds: α-3-O- and α-4-O-glycosylation products **13** and **14**, which were identified by NMR spectroscopy. In particular, the formation of α-glycoside bonds was confirmed by coupling constant *J*_1,2_ for the GalN-unit (3.8 and 3.6 Hz in ^1^H NMR spectra for **13** and **14**, respectively). Regioselectivity of the glycosylation reaction was confirmed by comparing the downfield signals H-3Gal and H-4Gal relative to each other in the ^1^H NMR spectra (**13**: 4.24 ppm for H-3Gal; 5.99 ppm for H-4Gal; **14**: 5.42 ppm for H-3Gal; 4.46 ppm for H-4Gal) and by downfield signals C-3Gal of **13** (73.2 ppm) and C-4Gal of **14** (74.5 ppm) in the ^13^C NMR spectra.

**Scheme 4:**
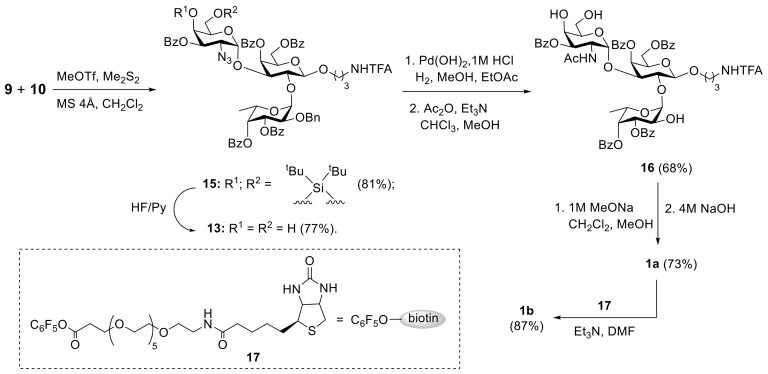
Assembly of the A trisaccharide backbone and preparation of target compound **1a** and its biotinylated derivative **1b**.

As an alternative method to conduct 3-O-glycosylation with donor **10,** we used monohydroxy-acceptor **9** (Scheme 4). As expected, the coupling of compounds **9** and **10** was stereoselective and gave the desired trisaccharide **15** in an 81% yield, contaminated by traces of isomeric product that was formed due to the migration of a benzoyl group in **9** from O-4 to O-3 in the galactose unit during the reaction. Further removal of the di-tert-butylsilylene-group by HF/Py solution and chromatography purification gave the individual diol **13**. The α-configuration of the glycoside bond at the GalN-unit of **13** was confirmed by the characteristic value of the corresponding coupling constant *J*_1,2_ (3.8 Hz) in the ^1^H NMR spectrum. Hydrogenolysis of **13** to remove the 2-O-benzyl group at the fucosyl residue and reduce the azide substituent to an amine and subsequent N-acetylation resulted in the formation of trisaccharide **16** in an overall yield of 68%. Its saponification gave the target spacered A trisaccharide **1a** (73%), which was then treated with the biotin derivative bearing an activated ester group **17** [33] to give the glycoconjugate **1b**.

## 3. Materials and Methods

### 3.1. General Information

All reagents were purchased at Sigma-Aldrich unless otherwise noted. MeCN and CH_2_Cl_2_ were distilled over P_2_O_5_ and CaH_2_. MeOH was distilled over Mg(OMe)_2_. Anhydrous pyridine and DMF were commercial (Sigma-Aldrich). Molecular sieves AW-300 MS (4Å) were crushed and activated before reaction for 5 min at 400–500 °C in vacuo. Amberlite IR-120 (hydrogen form, Fluka) was washed with 1M aq. HCl, H_2_O, acetone, and dried.

Thin-layer chromatography (TLC) was carried out on aluminum sheets coated with silica gel 60 F254 (Merck). TLC plates were inspected under UV light (λ = 254 nm) and developed with treatment by a mixture of 15% H_3_PO_4_ and orcinol (1.8 g/L) in EtOH–H_2_O (5: 95, *v*/*v*) followed by heating. Flash chromatography was performed on a Buchi Reveleris X2 system using Buchi FlashPure EcoFlex cartridges (irregular 40–63 μm silica). Column chromatography was performed with silica gel 60 (40–63 μm, E. Merck). Gel-filtration was performed on a TSK-40 HW(S) column (420 × 25 mm) and Sephadex G-15 column (400 × 17 mm) by elution with 0.1 M AcOH in water at a flow rate of 0.5 mL·min^−1^ with a RI detector.

NMR spectra were recorded on Bruker Fourier 300HD (300 MHz), Bruker AV400 (400 MHz), or Bruker AV600 (600 MHz) spectrometers at temperatures denoted on the spectra. The resonance assignment in ^1^H and ^13^C NMR spectra was performed using 2D-experiments (COSY, HSQC). Chemical shifts are reported in ppm referenced to tetramethylsilane as a standard for 1H and solvent signal (δ = 77.16 for CDCl_3_) for ^13^C. ^1^H-NMR spectra in D_2_O were registered with water suppression using a presaturation pulse sequence. See all NMR spectra in Supplementary Materials.

High-resolution mass spectra (HRMS) were recorded on a Bruker micrOTOF II instrument using electrospray ionization (ESI). The measurements were performed in positive ion mode (interface capillary voltage −4500 V) or in negative ion mode (3200 V); mass range from *m*/*z* 50 to *m*/*z* 3000 Da; external or internal calibration was made with an electrospray calibrant solution (Fluka). A syringe injection was used for solutions in a mixture of acetonitrile and water (50:50 *v*/*v*, flow rate 3 μL·min^−1^). Nitrogen was applied as a dry gas; interface temperature was set at 180 °C.

Glycosylation reactions were carried out in anhydrous solvent. Powdered molecular sieves were activated for 2 h at 180 °C in vacuo using an oil pump before use in the reaction.

### 3.2. Synthesis of Compounds **4**, **8**, **9**, **13, 14, 1a** and **1b**

#### 3.2.1. 3-Trifluoroacetamidopropyl 3,4-*O*-Isopropylidene-6-*O*-Benzoyl-β-D-Galactopyranoside (**4**)

To a stirred solution of galactoside **2** (312.5 mg, 0.94 mmol) in collidine (1.4 mL), BzCl (120 µL, 1.03 mmol, 1.1 eq.) was added. The mixture was stirred at 0 °C for 2 h, quenched with dimethylaminopropylamine (DMAPA), diluted with EtOAc, washed with 1 M aq. HCl, sat. aq. NaHCO_3_, the combined organic phase was dried by filtration through Na_2_SO_4_, and concentrated in vacuo. The residue was purified by flash chromatography (CH_2_Cl_2_:MeOH (0→8%), giving 291.9 mg (71%) of benzoylated monosaccharide **3**. It was dissolved on 2,2-dimethoxypropane (5 mL) and CSA (73.5 mg) was added. The mixture was stirred at RT for 1.5 h, quenched with Et_3_N, and co-evaporated with toluene in vacuo. The residue was purified by flash chromatography (toluene:ethyl acetate 20→50%), giving 286.8 mg (90%; summary yield 64%) of monosaccharide **4**. [α]*_D_*^16^ + 29.5 (*c* 1, CHCl_3_). ^1^H NMR (400 MHz, CDCl_3_): 1.36 and 1.53 (both s, on 3H, 2 CH_3_), 1.82–1.91 (m, 2H, OCH_2_CH*_2_*CH_2_N), 2.44 (s, 1H, OH), 3.49 (qd, 2H, *J* = 2.7 Hz, 6.1 Hz; OCH_2_CH_2_C*H_2_*N), 3.57 (t, 1H, *J* = 7.7 Hz; H-2), 3.68–3.76, and 3.92–3.98 (both m, on 1H, OCH_2_CH_2_CH_2_N), 4.09–4.13 (m, 1H, H-3), 4.13–4.18 (m, 1H, H-5), 4.21–4.24 (m, 2H, H-1, H-4), 4.60–4.63 (m, 2H, H-6), 7.42–8.05 (m, 5H, Ph). ^13^C NMR (100 MHz, CDCl_3_): 26.3, 28.0 (2 CH_3_), 28.3 (OCH_2_*C*H_2_CH_2_N), 38.3 (OCH_2_CH_2_*C*H_2_N), 63.6 (C-6), 69.0 (O*C*H_2_CH_2_CH_2_N), 71.4 (C-5), 73.3 (C-4), 73.5 (C-2), 79.0 (C-3), 102.4 (C-1), 110.7 (CMe_2_), 128.5, 129.6, 133.3, 166.3 (Ph), 166.3 (COPh). HRMS (ESI) *m/z*: found [M + NH_4_]^+^ 495.1941, C_21_H_26_F_3_NO_8_ calcd for [M + NH_4_]^+^ 495.1949.

#### 3.2.2. 3-Trifluoroacetamidopropyl 2-*O*-Benzyl-3,4-Di-*O*-Benzoyl-α-L-Fucopyranosyl- (1→2)-6-*O*-Benzoyl-β-D-Galactopyranoside (**8**)

A carefully dried mixture of donor **5** (389.8 mg, 0.64 mmol, 1.45 eq.) and galactosyl acceptor **4** (211.6 mg, 0.44 mmol, 1 eq.) was dissolved in CH_2_Cl_2_ (6 mL) and molecular sieves 4 Å (600 mg) were added. The mixture was cooled to −30 °C and TMSOTf (8 µL, 44 µmol, 0.1 eq.) was added. The mixture was stirred and warmed up to ambient temperature for 3 h and then quenched with one drop of Et_3_N. The mixture was filtered through a Celite pad with CH_2_Cl_2_, and the filtrate was evaporated in vacuo. The residue was purified by flash chromatography (toluene:ethyl acetate 0→10%), giving 369.2 mg (91%) of disaccharide **6** with inseparable minor quantity (~11%) of β-isomer **7**. ^1^H NMR (600 MHz, CDCl_3_, inter alia): 4.38 (d, 1H, *J* = 8.0 Hz; H-1Gal of **7**), 4.43 (d, 1H, *J* = 8.2 Hz; H-1Gal of **6**), 4.99 (d, 1H, *J*_1,2_ = 8.02 Hz; H-1Fuc of **7**), 5.63 (d, 1H, *J* = 3.4 Hz; H-1Fuc of **6**). ^13^C NMR (150 MHz, CDCl_3_, inter alia): 95.5 (C-1Fuc of **6**), 101.3 (H-1Gal of **6**), 102.7 (H-1Gal of **7**), and 103.3 (C-1Fuc of **7**).

To the disaccharide mixture (369.2 mg, 0.4 mmol) in CH_2_Cl_2_ (4 mL) was added 90% aq. TFA (400 µL). After 15 min, the mixture was diluted with CH_2_Cl_2_, washed with sat. aq. NaHCO_3_, the organic phase was dried by filtration through Na_2_SO_4_ and concentrated in vacuo. The residue was purified by flash chromatography (CHCl_3_: acetone 0→10%), giving 278 mg (79%; summary yield 72%) of disaccharide **8**. [α]*_D_*^17^ −95.1 (*c* 1, CHCl_3_). ^1^H NMR (600 MHz, CDCl_3_): 1.18 (d, 3H, *J* = 6.5 Hz; CH_3_-Fuc), 1.83–1.97 (m, 2H, OCH_2_C*H_2_*CH_2_N), 2.73 (s, 1H, OH-4Gal), 3.39–3.46, and 3.53–3.60 (both m, on 1H, OCH_2_CH_2_C*H_2_*N), 3.64–3.73 (m, 2H, OC*H*HCH_2_CH_2_N, H-2Gal), 3.76 (dd, 1H, *J* = 3.0; 9.0 Hz; H-3Gal), 3.86 (t, 1H, *J* = 6.4 Hz; H-5Gal), 3.99–4.05 (m, 2H, OCH*H*CH_2_CH_2_N, H-4Gal), 4.18 (dd, 1H, *J* = 3.5; 10.3 Hz; H-2Fuc), 4.33 (s, 1H, OH-3Gal), 4.38 (d, 1H, *J* = 7.8 Hz; H-1Gal), 4.50 (q, 1H, *J* = 6.6 Hz; H-5Fuc), 4.59–4.62 (m, 1H, H-6Gal), 4.64 and 4.72 (AB system, *J* = 11.4 Hz; C*H_2_*-Ph), 5.16 (d, 1H, *J* = 3.6 Hz; H-1Fuc), 5.65 (d, 1H, *J* = 3.3 Hz; H-4Fuc), 5.74 (dd, 1H, *J* = 3.2; 10.3 Hz; H-3Fuc), 7.24–7.64 (m, 20H, 4 Ph). ^13^C NMR (150 MHz, CDCl_3_): 16.1 (C-6Fuc), 28.4 (OCH_2_*C*H_2_CH_2_N), 38.6 (OCH_2_CH_2_*C*H_2_N), 63.0 (C-6Gal), 66.1 (C-5Fuc), 67.9 (C-4Gal), 68.8 (O*C*H_2_CH_2_CH_2_N), 71.0 (C-3Fuc), 72.0 (C-4Fuc), 72.3 (C-5Gal), 73.1 (C-3Gal), 74.2 (*C*H_2_-Ph), 74.3 (C-2Fuc), 81.9 (C-2Gal), 100.6 (C-1Fuc), 102.0 (C-1Gal), 128.3–136.3 (4 Ph), 157.1 (COCF_3_), 165.7, 165.8, 166.3 (3 COPh). HRMS (ESI) *m/z*: found [M + Na]^+^ 904.2764, C_45_H_46_F_3_NO_14_ calcd for [M + Na]^+^ 904.2763.

#### 3.2.3. 3-Trifluoroacetamidopropyl 2-*O*-Benzyl-3,4-di-*O*-Benzoyl-α-L-Fucopyranosyl- (1→2)-4,6-Di-*O*-Benzoyl-β-D-Galactopyranoside (**9**)

To a stirred solution of disaccharide **8** (278 mg, 0.32 mmol) in CH_3_CN (3 mL) was added trimethyl orthobenzoate (181 µL, 1.06 mmol, 3.3 eq.) and catalytic amounts of CSA (15 mg) up to pH <7. At the end of the reaction according to TLC, H_2_O (50 µL) was added. The mixture was diluted with CH_2_Cl_2_, washed with sat. aq. NaHCO_3_, the organic phase was dried by filtration through Na_2_SO_4_ and concentrated in vacuo. The residue was purified by flash chromatography (toluene:ethyl acetate 0→20%), giving 294.3 mg (93%) of disaccharide **9**. [α]*_D_*^28^ −92.3 (*c* 1, CHCl_3_). ^1^H NMR (400 MHz, CDCl_3_): 1.19 (d, 3H, *J* = 6.5 Hz; CH_3_-Fuc), 1.91–2.00 (m, 2H, OCH_2_C*H_2_*CH_2_N), 3.49–3.57 (m, 2H, OCH_2_CH_2_C*H_2_*N), 3.71–3.77 (m, 1H, H-2Gal), 3.80–3.87 (m, 1H, OC*H*HCH_2_CH_2_N), 4.01–4.09 (m, 2H, OCH*H*CH_2_CH_2_N, H-3Gal), 4.10–4.17 (m, 2H, H-5Gal_,_ H-2Fuc), 4.40 (dd, 1H, *J* = 5.8, 11.28 Hz; H-6aGal), 4.49–4.67 (m, 5H, H-5Fuc, H-6bGal, H-1Gal, C*H_2_*-Ph), 5.10 (d, 1H, *J* = 3.6 Hz; H-1Fuc), 5.66 (d, 1H, *J* = 3.3 Hz; H-4Fuc), 5.73 (dd, 1H, *J* = 3.2; 10.4 Hz; H-3Fuc), 5.77 (d, 1H, *J* = 3.4 Hz; H-4Gal), 7.03–8.18 (m, 25H, 5 Ph). ^13^C NMR (100 MHz, CDCl_3_): 16.1 (C-6Fuc), 28.3 (OCH_2_*C*H_2_CH_2_N), 39.0 (OCH_2_CH_2_*C*H_2_N), 62.3 (C-6Gal), 65.9 (C-5Fuc), 69.5 (C-4Gal), 69.7 (O*C*H_2_CH_2_CH_2_N), 71.0 (C-3Fuc), 71.73 (C-5Gal), 72.0 (C-4Fuc), 72.5 (C-3Gal), 73.5 (C-2Fuc), 73.9 (*C*H_2_-Ph), 81.7 (C-2Gal), 100.8 (C-1Fuc), 102.3 (C-1Gal), 128.3–136.4 (5Ph), 165.6, 165.8, 165.9, 166.1 (4 COPh). HRMS (ESI) *m*/*z*: found [M + NH_4_]^+^ 1003.3472, C_52_H_50_F_3_NO_15_ calcd for [M + NH_4_]^+^ 1003.3471.

#### 3.2.4. 3-Trifluoroacetamidopropyl 2-Azido-3-O-Benzoyl-2-Deoxy-α-D-Galactopyranosyl- (1→3)-[2-*O*-Benzyl-3,4-Di-*O*-Benzoyl-α-L-Fucopyranosyl-(1→2)]-4,6-Di-*O*-Benzoyl-β-D-Galactopyranoside (**13**) and 3-Trifluoroacetamidopropyl 2-Azido-3-O-Benzoyl-2-Deoxy-α-D-Galactopyranosyl-(1→4)-[2-*O*-Benzyl-3,4-Di-*O*-Benzoyl-α-L-Fucopyranosyl-(1→2)]- 4,6-Di-*O*-Benzoyl-β-D-Galactopyranoside (**14**)

**A**: A carefully dried mixture of donor **10** (29.4 mg, 50 µmol, 1.1 eq.) and disaccharide acceptor **8** (39.7 mg, 45 µmol) was dissolved in CH_2_Cl_2_ (1 mL) and molecular sieves 4 Å (100 mg) were added. After 10 min, MeOTf (27 µL, 0.25 mmol, 5.5 eq.) and Me_2_S_2_ (22 µL,0.25 mmol, 7.5 eq.) were added. The mixture was stirred for 2 h at room temperature and quenched with one drop of Et_3_N. The mixture was filtered through a Celite pad with CH_2_Cl_2_ and the filtrate was washed with sat. aq. NaHCO_3_. The organic phase was separated, and the solvent was evaporated in vacuo. The residue was purified by flash chromatography (toluene:ethyl acetate 0→10%), giving 35.5 mg of an inseparable mixture of trisaccharides **11** and **12**. To a solution of purified mixture in Py (0.3 mL) was added BzCl (7 µL, 54 µmol, 2 eq.) while stirring. After 30 min, the mixture was diluted with EtOAc, washed with 1 M aq. HCl, sat. aq. NaHCO_3_, the combined organic phase was dried by filtration through Na_2_SO_4_, and concentrated in vacuo. The residue was purified by flash chromatography (toluene:ethyl acetate 0→10%). To a solution of the obtained compound in Py (0.5 mL) was added 40% aq. HF (83 µL). After 10 min, the mixture was diluted with EtOAc, washed with 1 M aq. HCl, sat. aq. NaHCO_3_, the combined organic phase was dried by filtration through Na_2_SO_4_ and concentrated in vacuo. The residue was purified by flash chromatography (toluene: ethyl acetate 20→50%), giving 19.3 mg (34%) of trisaccharide **13** and 10.3 mg (19%; purity 85%) of trisaccharide **14**. **13**: [α]*_D_*^20^ +10.4 (*c* 1, CHCl_3_). ^1^H NMR (400 MHz, CDCl_3_): 1.21 (d, 3H, *J* = 6.4 Hz; CH_3_-Fuc), 1.92–1.99 (m, 2H, OCH_2_ C*H_2_*CH_2_N), 3.41–3.60 (m, 2H, OCH_2_CH_2_C*H_2_*N), 3.72–3.85 (m, 3H, H-6aGalN, OC*H*HCH_2_CH_2_N), 3.89 (dd, 1H, *J* = 3.7; 10.9 Hz, H-2GalN), 4.01–4.09 (m, 2H, H-2Gal, OCH*H*CH_2_CH_2_N), 4.10–4.19 (m, 2H, H-5Gal, H-2Fuc), 4.24 (dd, 1H, *J* = 3.4; 9.6 Hz, H-3Gal), 4.32 (d, 1H, *J* = 2.8 Hz; H-4GalN), 4.35–4.41 (m, 2H, H-5GalN, H-6aGal), 4.55–4.65 (m, 3H, H-1Gal, H-5Fuc, H-6bGal), 4.72–4.82 (m, 2H, C*H_2_*-Ph), 5.26 (d, 1H, *J* = 3.7 Hz, H-1Fuc), 5.44 (dd, 1H, *J* = 2.9; 10.9 Hz; H-3GalN), 5.54 (d, 1H, *J* = 3.8 Hz, H-1GalN), 5.61–5.67 (m, 2H, H-4Fuc, H-3Fuc), 5.99 (d, 1H, *J* = 3.4 Hz, H-4Gal), 7.05–8.20 (m, 30H, 6Ph). ^13^C NMR (100 MHz, CDCl_3_): 16.2 (C-6Fuc), 28.5 (OCH_2_*C*H_2_CH_2_N), 38.4 (OCH_2_CH_2_ *C*H_2_N), 57.3 (C-2GalN), 62.17 (C-6Gal), 62.9 (C-6GalN), 64.6 (C-4Gal), 65.6 (C-5Fuc), 68.52 (C-4GalN), 69.1 (O*C*H_2_CH_2_CH_2_N), 70.1 (C-5GalN), 70.9 (C-5Gal, C-3Fuc), 71.4 (C-3GalN), 72.1 (C-4Fuc), 73.0 (C-2Fuc), 73.2 (C-3Gal), 73.5 (***C***H_2_-Ph), 77.4 (C-2Gal), 93.5 (C-1GalN), 99.1 (C-1Fuc), 103.3 (C-1Gal), 127.7–137.9(6Ph), 164.9, 165.9, 166.0, 166.1, 166.4 (5 COPh). **14**: ^1^H NMR (400 MHz, CDCl_3_): 1.21 (d, 3H, *J* = 6.5 Hz; CH_3_-Fuc), 1.95–2.00 (m, 2H, OCH_2_C*H_2_*CH_2_N), 3.33 (dd, 1H, *J* = 4.1; 12.2 Hz; H-6aGalN), 3.39 (dd, 1H, *J* = 4.5; 12.2 Hz; H-6bGalN), 3.44–3.60 (m, 2H, OCH_2_CH_2_C*H_2_*N), 3.79–3.85 (m, 1H, OC*H*HCH_2_CH_2_N), 4.00–4.20 (m, 8H, OCH*H*CH_2_CH_2_N, H-2GalN, H-2Gal, H-2Fuc, H-5Gal, H-5GalN, C*H_2_*-Ph), 4.44 (d, 1H, *J* = 3.0 Hz; H-4GalN), 4.46 (d, 1H, *J* = 2.9 Hz; H-4Gal), 4.66–4.80 (m, 4H, H-1Gal, H-5Fuc, H-6Gal), 5.14 (d, 1H, *J* = 3.6 Hz; H-1GalN), 5.42 (dd, 1H, *J* = 2.9; 10.1 Hz; H-3Gal), 5.50 (dd, 1H, *J* = 2.9; 11.0 Hz; H-3GalN), 5.54 (d, 1H, *J* = 3.5 Hz; H-1Fuc), 5.60–5.67 (m, 2H, H-4Fuc, H-3Fuc), 6.77–8.20 (m, 30H, 6Ph). ^13^C NMR (100 MHz, CDCl_3_): 15.9 (C-6Fuc), 28.5 (OCH_2_*C*H_2_CH_2_N), 37.8 (OCH_2_CH_2_*C*H_2_N), 58.3 (C-2GalN), 62.2 (C-6Gal), 63.0 (C-6GalN), 65.6 (C-5Fuc), 67.7 (O*C*H_2_CH_2_CH_2_N), 68.9 (C-4GalN), 69.7 (C-5GalN), 70.3 (C-3Fuc), 71.9 (C-3GalN), 72.1 (C-4Fuc), 72.2 (*C*H_2_-Ph), 72.3 (C-5Gal), 72.6 (C-2Fuc), 74.5 (C-4Gal), 75.8 (C-3Gal), 97.2 (C-1Fuc), 99.3 (C-1GalN), 102.3 (C-1Gal), 127.6-133.9 (6Ph), 165.5, 165.9 (2 *C*OPh). HRMS (ESI) *m/z*: found [M + NH_4_]^+^ 1294.4324, C_65_H_63_F_3_N_4_O_20_ calcd for [M + NH_4_]^+^ 1294.4326.

**B**: A carefully dried mixture of donor **10** (84.8 mg, 0.14 mmol, 1.2 eq.) and disaccharide acceptor **9** (118 mg, 0.12 mmol) was dissolved in CH_2_Cl_2_ (2 mL) and molecular sieves 4 Å (200 mg) were added. After 10 min, MeOTf (98.7 µL, 0.9 mmol, 7.5 eq.) and Me_2_S_2_ (79.7 µL,0.9 mmol, 7.5 eq.) were added. The mixture was stirred for 2 h at room temperature and quenched with one drop of Et_3_N. The mixture was filtered through a Celite pad with CH_2_Cl_2_ and the filtrate was washed with sat. aq. NaHCO_3_. The organic phase was separated, and the solvent was evaporated in vacuo. The residue was purified by flash chromatography (toluene:ethyl acetate 0→20%), giving 137.6 mg (81%) of trisaccharide **15** (contaminated by traces of (1→4)-glycosylation product). To a solution of the purified compound in Py (2 mL) was added 40% aq. HF (333 µL). After 10 min, the mixture was diluted with EtOAc, washed with 1 M aq. HCl, sat. aq. NaHCO_3_, the combined organic phase was dried by filtration through Na_2_SO_4_, and concentrated in vacuo. The residue was purified by flash chromatography (toluene:ethyl acetate 20→50%), giving 117.9 mg (77%; summary yield 62%) of trisaccharide **13**. All spectral characteristics w completely identical to the compound **13** obtained by method **A**.

#### 3.2.5. 3-Aminopropyl 2-Acetamido-2-Deoxy-α-D-Galactopyranosyl-(l→3)-[(α-L-Fucopyranosyl)-(l→2)]-β-D-Galactopyranoside (**1a**)

To a solution of the trisaccharide **13** (83.8 mg, 66 µmol) in EtOAc (2 mL) and MeOH (1mL) were added 1 M HCl (50 µL) and Pd(OH)_2_/C (100 mg) after the flask was filled with hydrogen. The reaction mixture was stirred for 3 h at RT. Then, the reaction mixture was filtered on a glass filter through a Celite pad and concentrated in vacuo. The crude material was dissolved in CHCl_3_:MeOH (2 mL in ratio 1:1), then Et_3_N (27 µL, 0.19 mmol) and Ac_2_O (12 µL, 0.13 mmol) were added. After completing the reaction, the mixture was concentrated in vacuo and purified by flash chromatography (CHCl_3_:MeOH 0→10%), giving 57.3 mg (68%) of trisaccharide **16**. One M MeONa (100 µL) was added to a solution of the purified compound in MeOH:CH_2_Cl_2_ (0.8 mL in ratio 3:1). The mixture was left for 2 h, then 4 M NaOH (50 µL) was added and left overnight. The base was neutralized by 1 M aq. HCl and the resulting solution was concentrated. The residue was purified by gel-permeation chromatography (TSK HW-40 (S), 0.1 M AcOH) giving 19.4 mg (73%) of trisaccharide **1a**. All NMR and HRMS data corresponded to the literature data [9].

#### 3.2.6. 3-(21-Biotinamino-4,7,10,13,16,19-Hexaoxagenicaminoamino)-Propyl 2-Acetamido-2-Deoxy-α-D-Galactopyranosyl-(l→3)-[(α-L-Fucopyranosyl)-(l→2)]-β-D-Galactopyranoside (**1b**)

To a solution of trisaccharide **1a** (0.5 mg, 0.85 μmol) in DMF (100 μL) was added a 0.0062 M solution of biotin-activated ester **17** (20 μL, 1.28 μmol, 1.5 eq.) and Et_3_N (15 μL, 0.1 mmol). After 20 min, the mixture was concentrated in vacuo, after which the residue was purified by gel-permeation chromatography (G-15, 0.1 M AcOH) to give 0.85 mg (87%) biotinylated conjugate **1b**. ^1^H NMR (600 MHz, D_2_O, characteristic signals): oligosaccharide fragment: 1.24 (d, 3H, *J* = 6.6 Hz; CH_3_-Fuc), 1.93 (s, 1H, NH*Ac*), 4.55 (d, 1H, *J* = 7.8 Hz; H-1Gal), 5.20 (d, 1H, *J* = 3.7 Hz; H-1GalN), 5.32 (d, 1H, *J* = 3.8 Hz; H-1Fuc); biotin fragment: 1.42–1.78 (m, 6H, H-b, H-c, H-d), 2.30 (t, 2H, *J* = 7.3 Hz; H-a), 2.81 (d, 1H, *J* = 13.0 Hz; H-h‘), 3.02 (dd, 1H, *J* = 5 Hz; 13.1 Hz; H-h), 3.34–3.38 (m, 1H, H-e), 4.63 (dd, 1H, *J* = 5.0 Hz; 7.9 Hz; H-g); linker: 1.83–1.89 (m, 2H, OCH_2_C*H_2_*CH_2_N), 2.54 (t, 2H, *J* = 6.1 Hz; OCH_2_CH_2_C*H_2_*N).^13^C NMR characteristic signals derived from (^1^H, ^13^C) HSQC spectrum (D_2_O): oligosaccharide fragment: 16.7 (*C*H_3_-Fuc), 26.4 (NH*Ac*), 92.7 (C-1GalN), 98.8 (C-1Fuc), 102.7 (C-1Gal); biotin fragment: 26.4 (C-c), 29.1 (C-b, C-d), 36.7 (C-a), 56.8 (C-e), 61.6 (C-g); linker: 29.8 (OCH_2_*C*H_2_CH_2_N), 37.4 (OCH_2_CH_2_*C*H_2_N). HRMS (ESI) *m/z*: found [M + Na]^+^ 1170.5197, C_48_H_85_N_5_O_24_S calcd for [M + Na]^+^ 1170.5178.

## 4. Conclusions

The synthesis of spacered A trisaccharide derivatives **1a** and **1b** was performed using a 2-azido-2-deoxy-selenogalactoside glycosyl donor bearing stereo-controlling 3-O-benzoyl and 4,6-O-(di-tert-butylsilylene)-protecting groups, showing once again the efficacy of this α-glycosylation agent for the assembly of even vicinally branched oligosaccharide chains. At the same time, we observed rather poor applicability of donor **10** for the regioselective glycosylation of diolic acceptor **8**. Obtained trisaccharide **1b** is being used in the coating of magnetic nanobeads for glycobiological applications to be described in due course.

## Data Availability

Data are available from the corresponding author.

## Supplementary Materials

The following are available online. Copies of NMR spectra of compounds **4**, **6**–**9**, **13**, **14**, **1a**, and **1b**.

## Sample Availability

Samples of described compounds are available from the corresponding author.
