# Supplementary material for: The Synthesis of Blood Group Antigenic A Trisaccharide and Its Biotinylated Derivative
Source: Molecules. 2021 Sep 28;26(19):5887. doi: 10.3390/molecules26195887 (PMC8512078; doi:10.3390/molecules26195887)

The Synthesis of Blood Group Antigenic A Trisaccharide and Its Biotinylated Derivative

Ekaterina D. Kazakova, Dmitry V. Yashunsky and Nikolay E. Nifantiev *

Laboratory of Glycoconjugate Chemistry, N. D. Zelinsky Institute of Organic Chemistry, Russian Academy of Sciences, Leninsky pr. 47, 119991 Moscow, Russia; edkazakova@gmail.com (E.D.K.); yashunsky1959@yandex.ru (D.V.Y.)
* Correspondence: nen@ioc.ac.ru

Table of Contents

Copies of NMR-spectra 2
^{1}H NMR of spacered A trisaccharide (**1a**) 2
^{13}C NMR of spacered A trisaccharide (**1a**) 2
^{1}H NMR of a biotinylated derivative of spacered A trisaccharide (**1b**) 3
(^{1}H, ^{13}C) HSQC NMR of a biotinylated derivative of spacered A trisaccharide (**1b**) 3
^{1}H NMR of compound **4** 4
^{13}C NMR of compound **4** 4
^{1}H NMR of mixture **6** and **7** (inter alia) 5
^{13}C NMR of mixture **6** and **7** (inter alia) 5
^{1}H NMR of compound **8** 6
^{13}C NMR of compound **8** 6
^{1}H NMR of compound **9** 7
^{13}C NMR of compound **9** 7
^{1}H NMR of compound **13** 8
^{13}C NMR of compound **13** 8
^{1}H NMR of compound **14** 9
^{13}C NMR of compound **14** 9

Copies of NMR-spectra

^{1}H NMR of spacered A trisaccharide (**1a**)

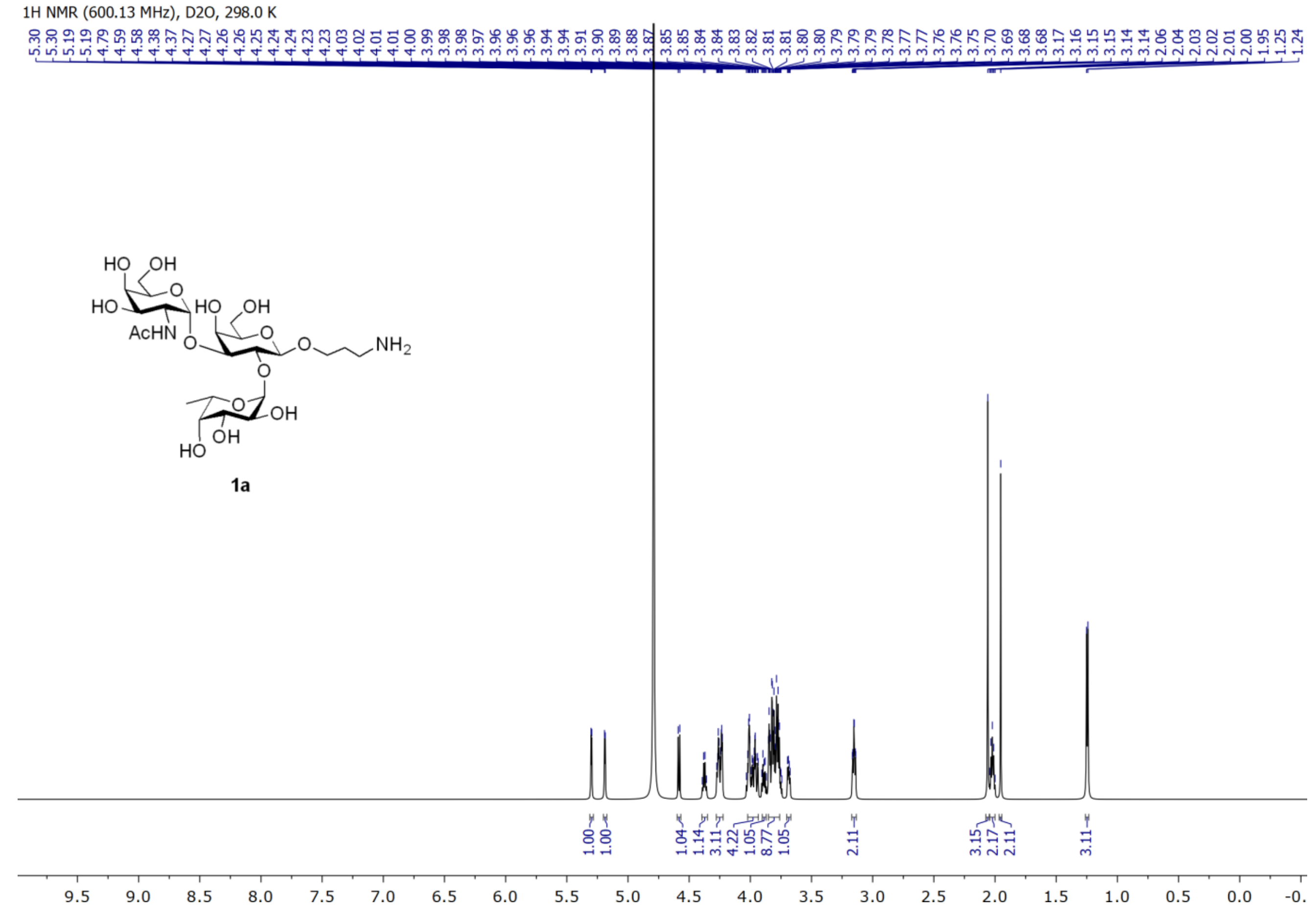

^{13}C NMR of spacered A trisaccharide (**1a**)

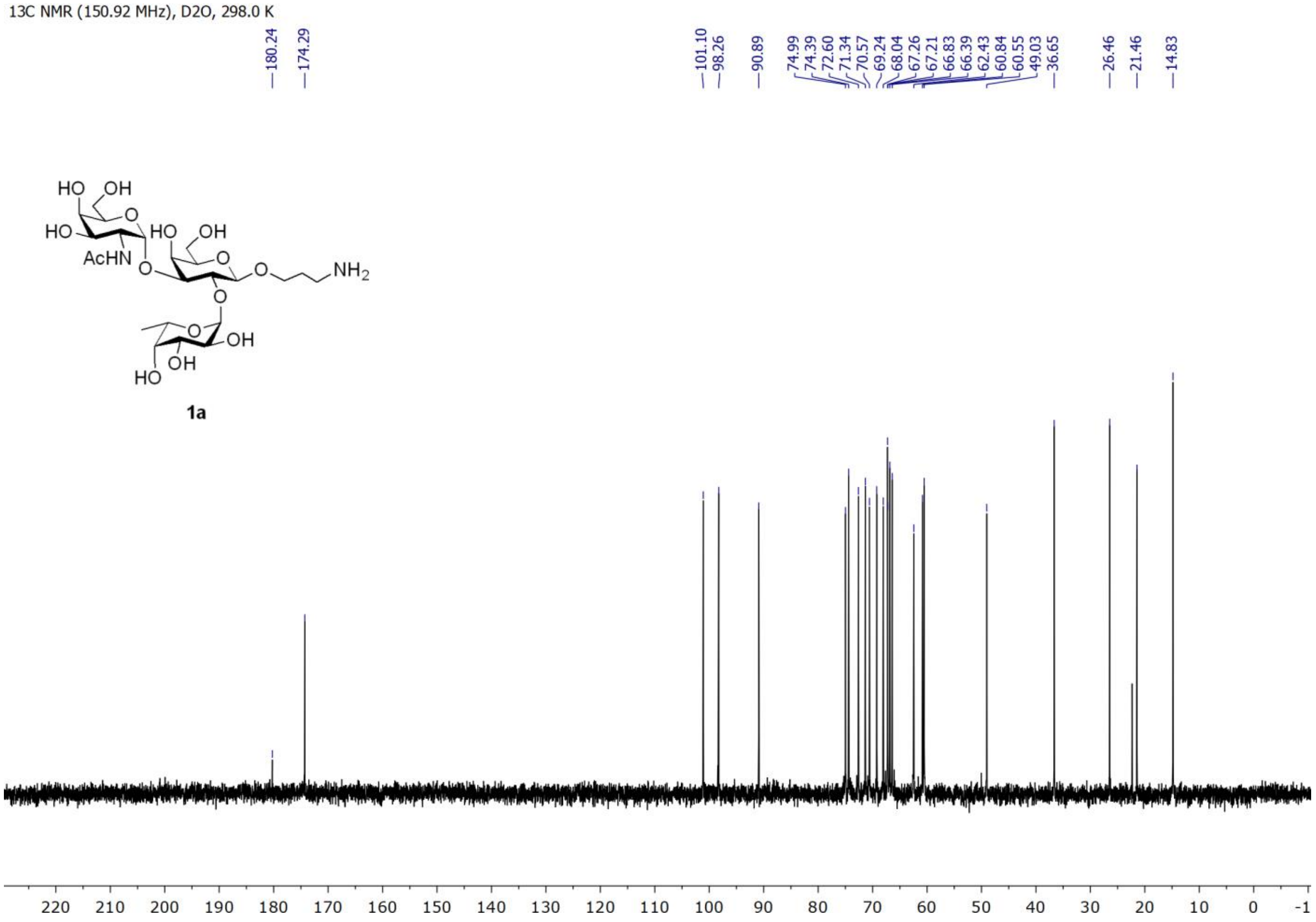

*^{1}H NMR of a biotinylated derivative of spacered A trisaccharide (**1b**)*

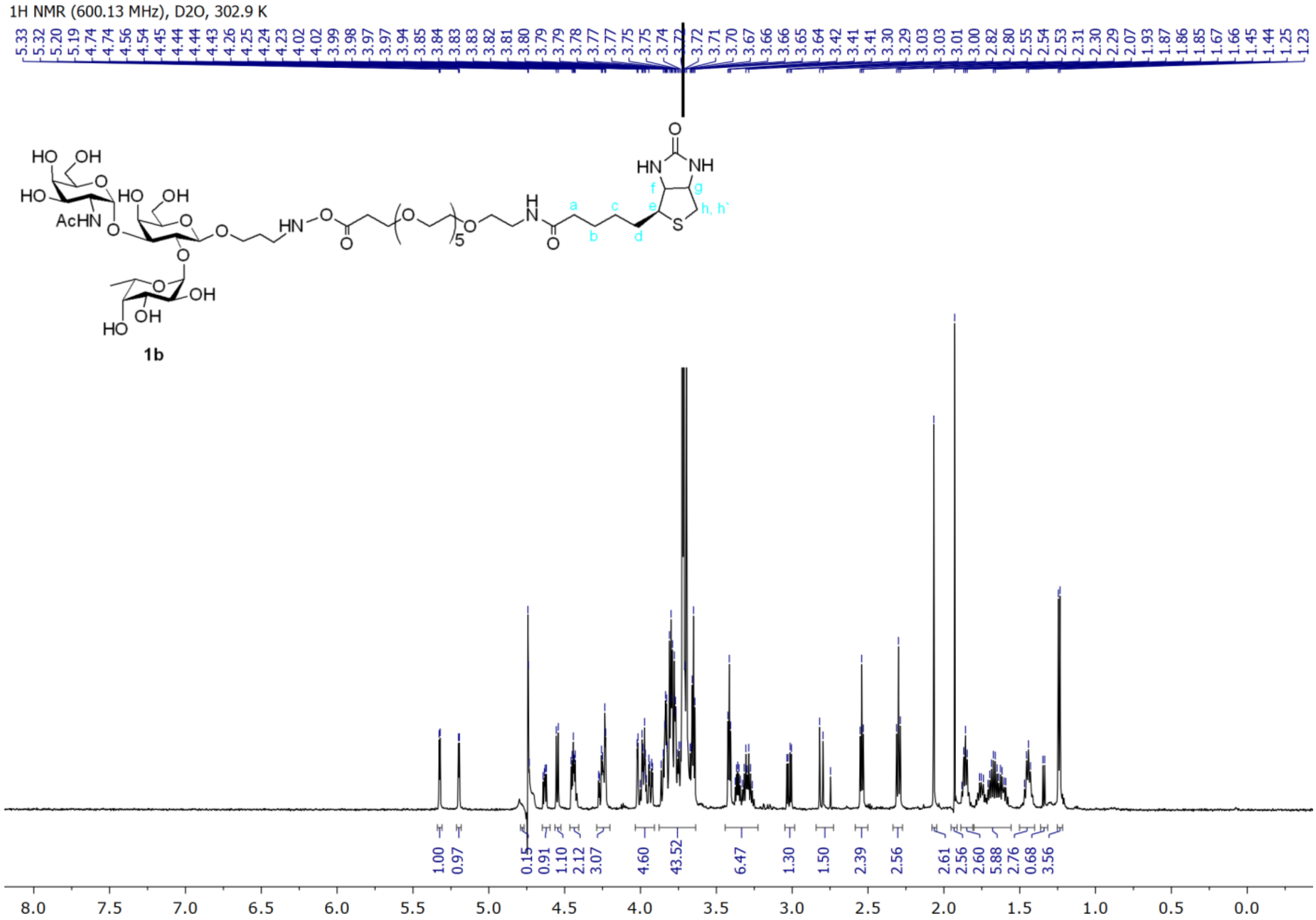

*(^{1}H, ^{13}C) HSQC NMR of a biotinylated derivative of spacered A trisaccharide (**1b**)*

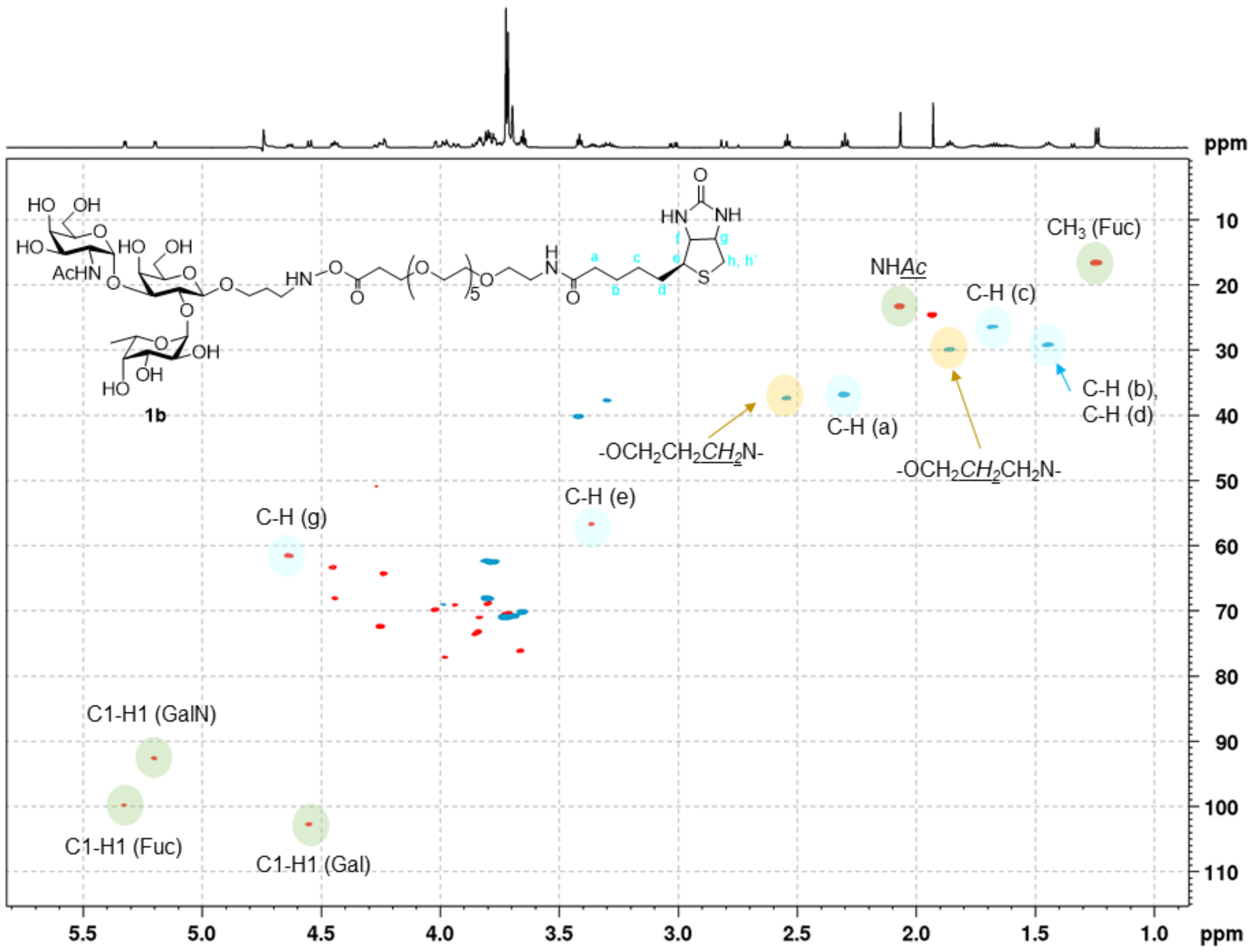

^{1}H NMR of compound **4**

1H NMR (400.16 MHz), CDCl3, 298.0 K

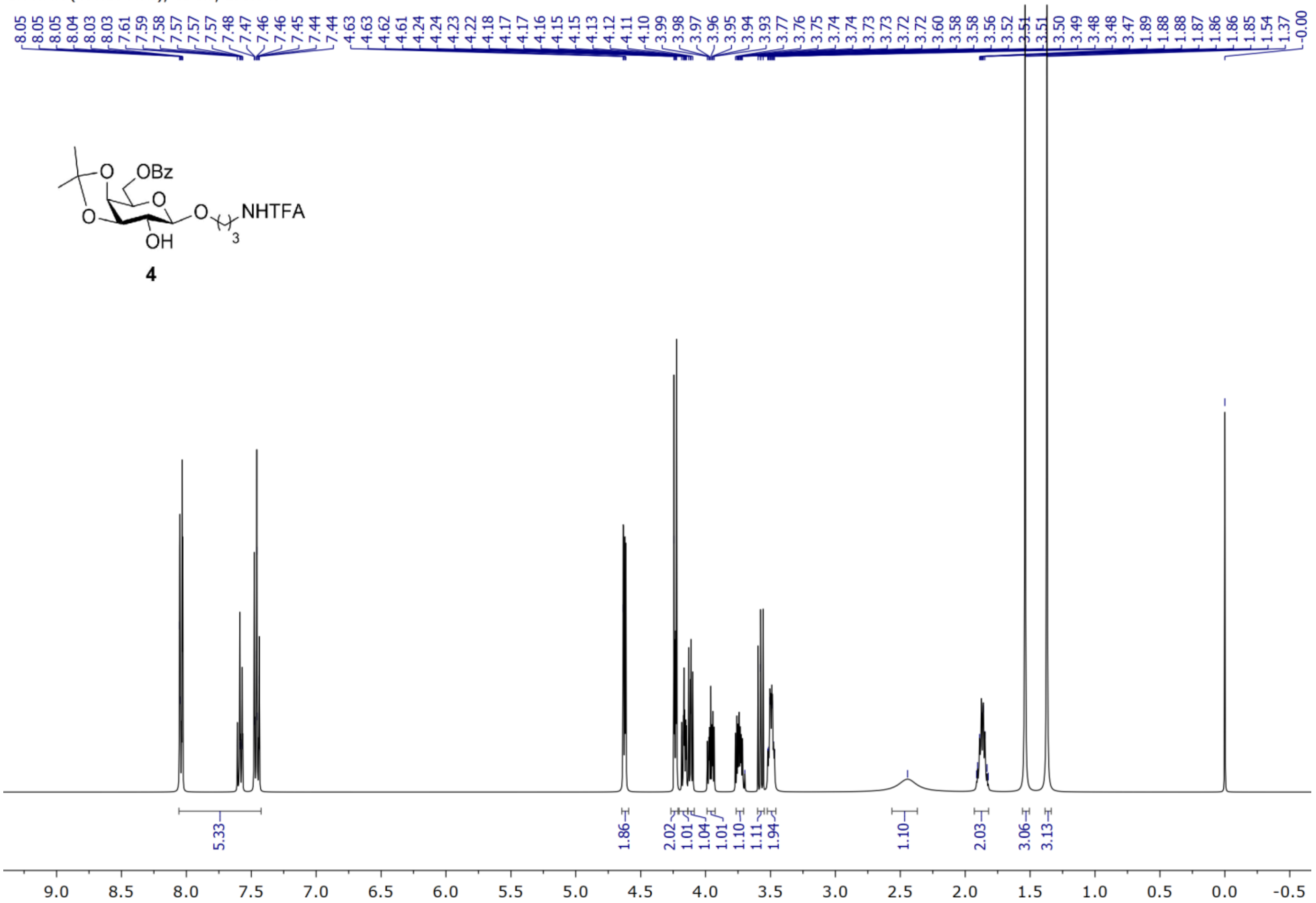

^{13}C NMR of compound **4**

13C NMR (100.63 MHz), CDCl3, 298.0 K

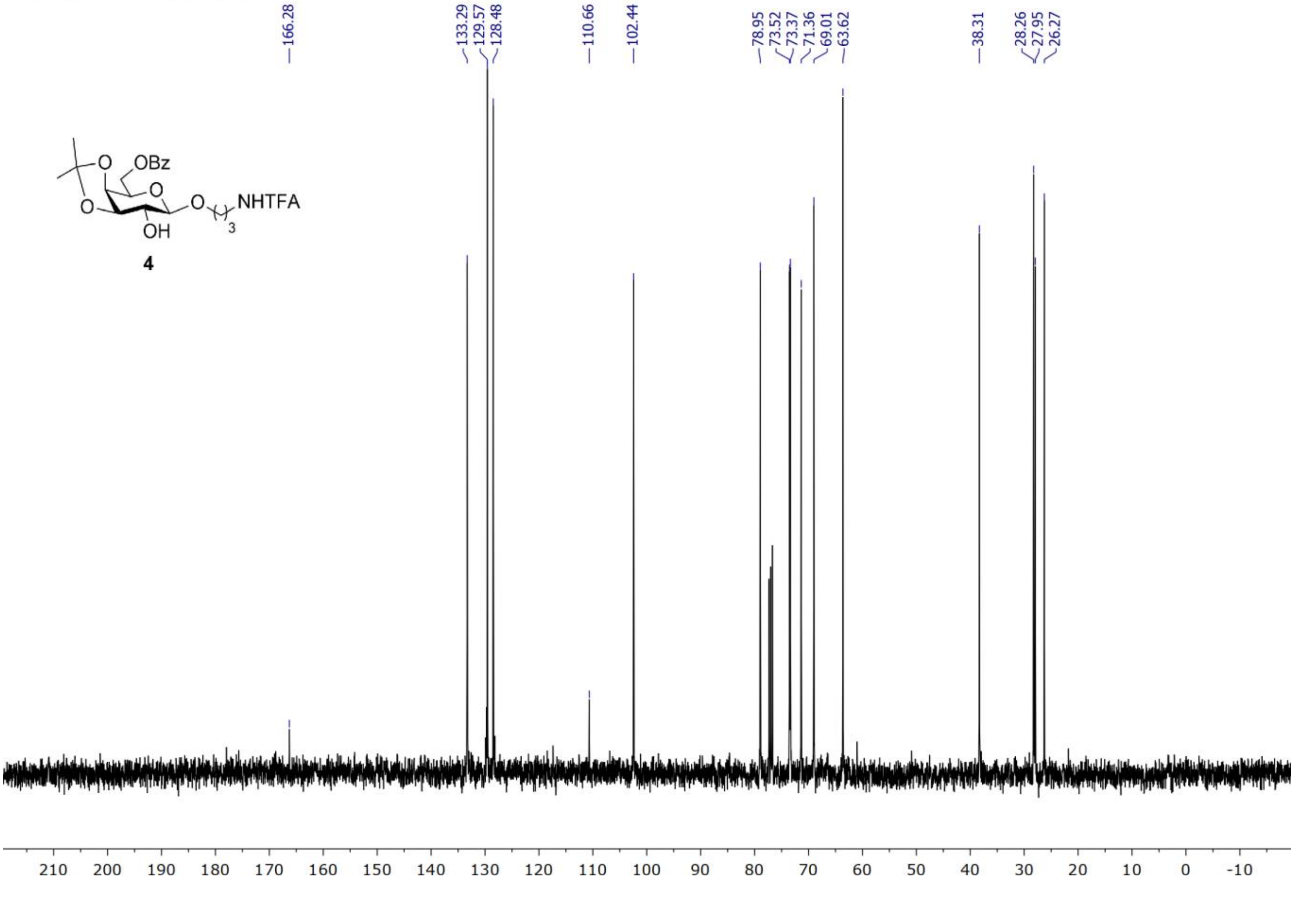

^{1}H NMR of mixture **6** and **7** (*inter alia)*

1H NMR (600.13 MHz), CDCl3, 298.8 K

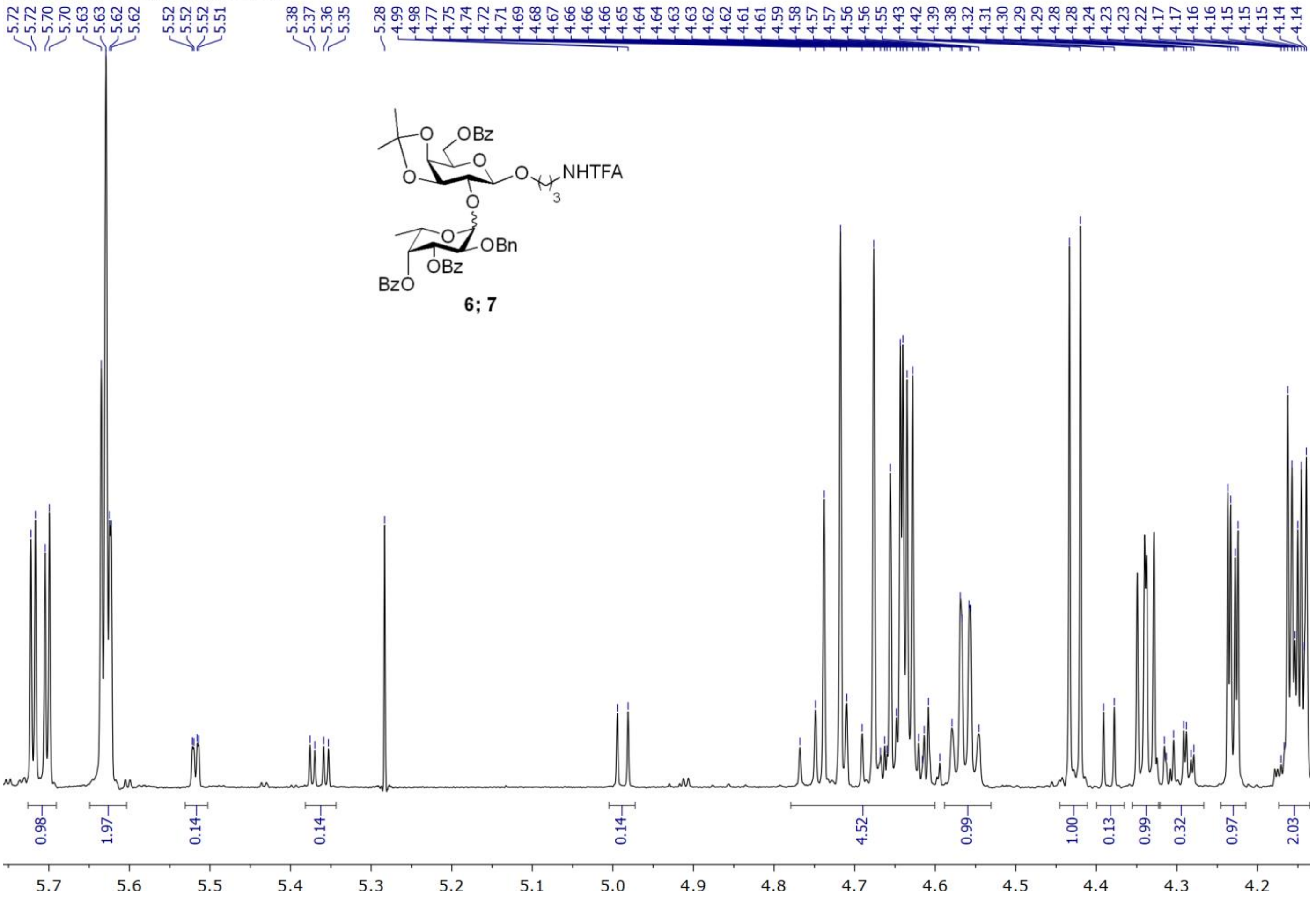

^{13}C NMR of mixture **6** and **7** (inter alia)

13C NMR (150.92 MHz), CDCl3, 298.8 K

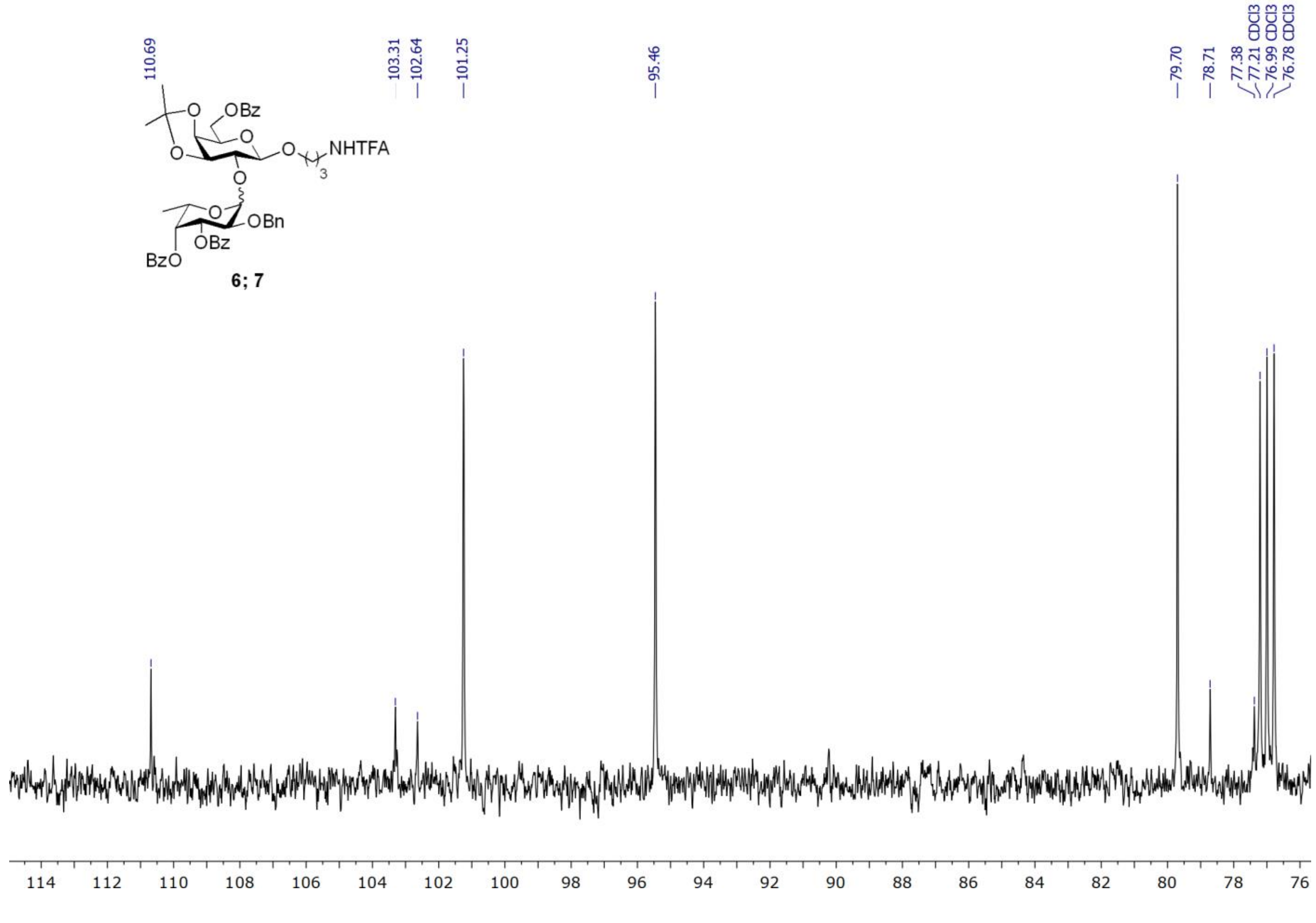

^{1}H NMR of compound **8**

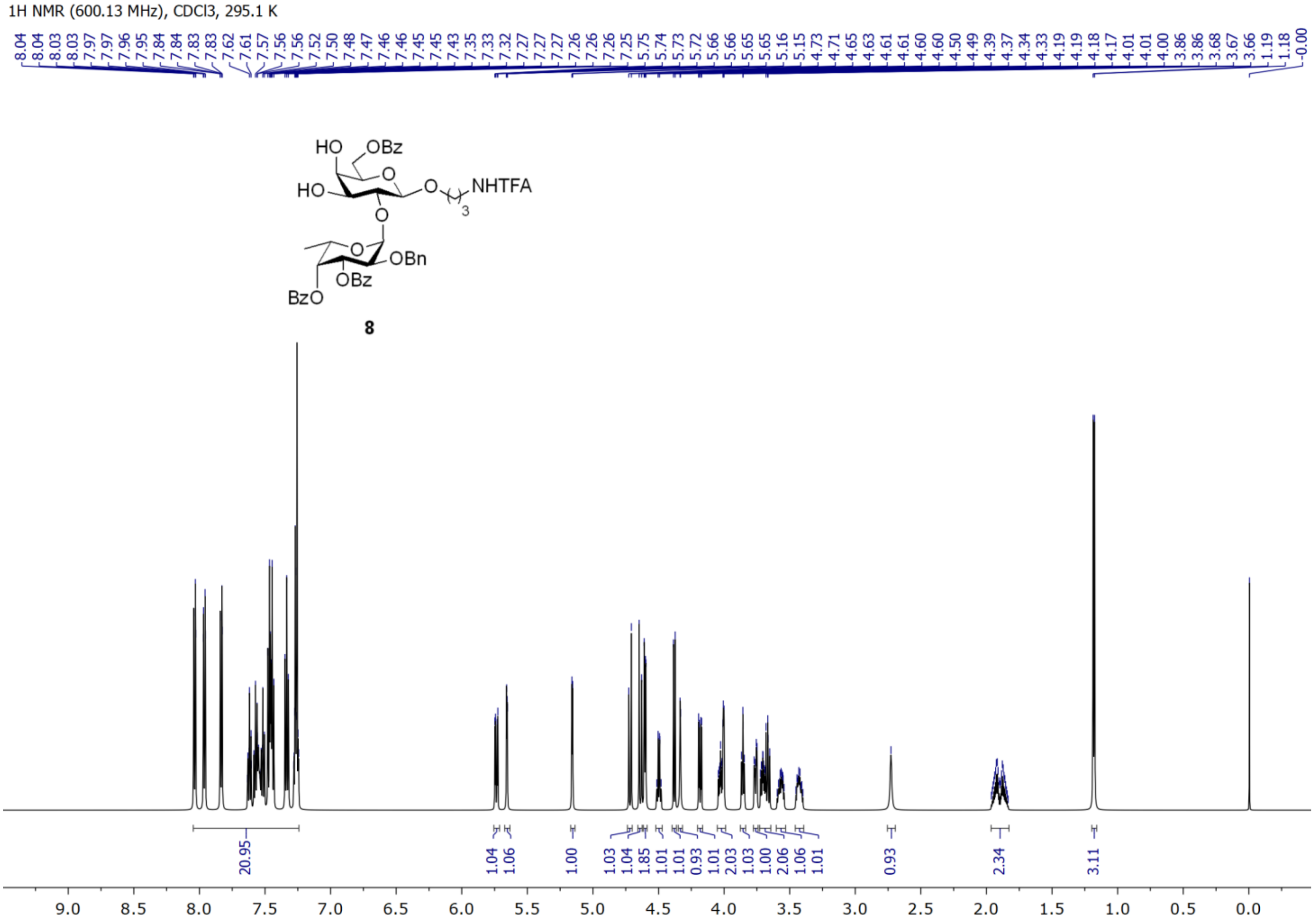

^{13}C NMR of compound **8**

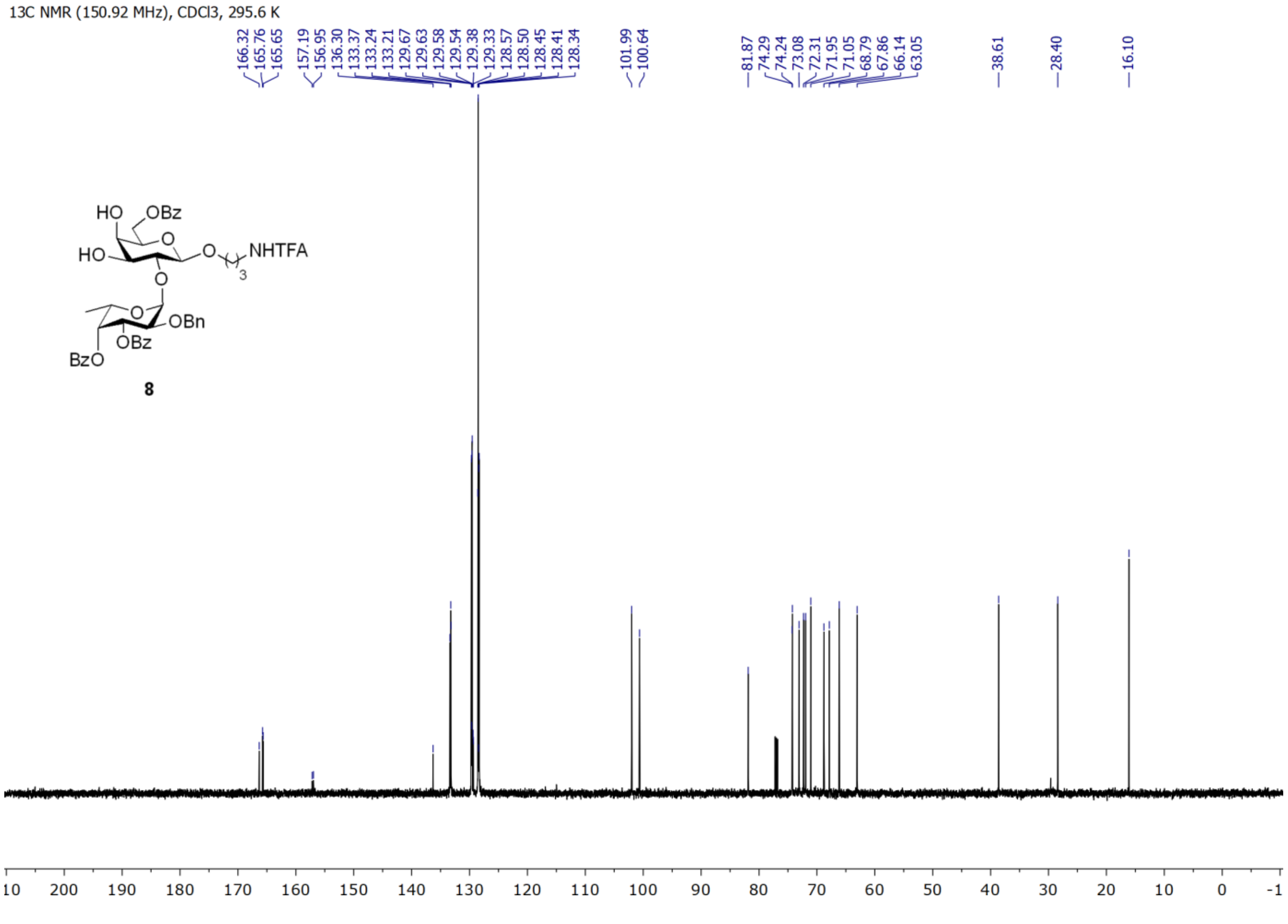

^{1}H NMR of compound **9**

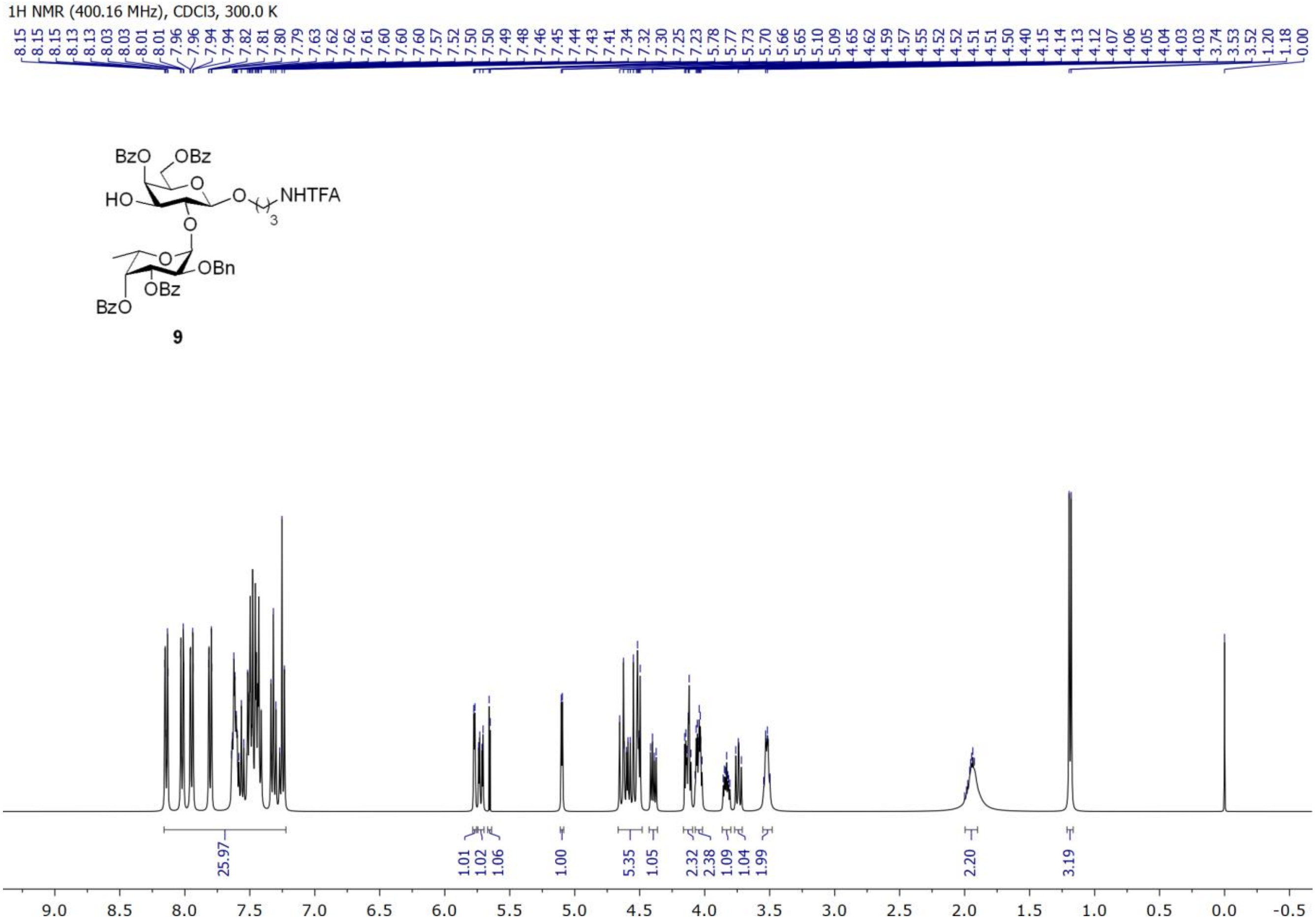

^{13}C NMR of compound **9**

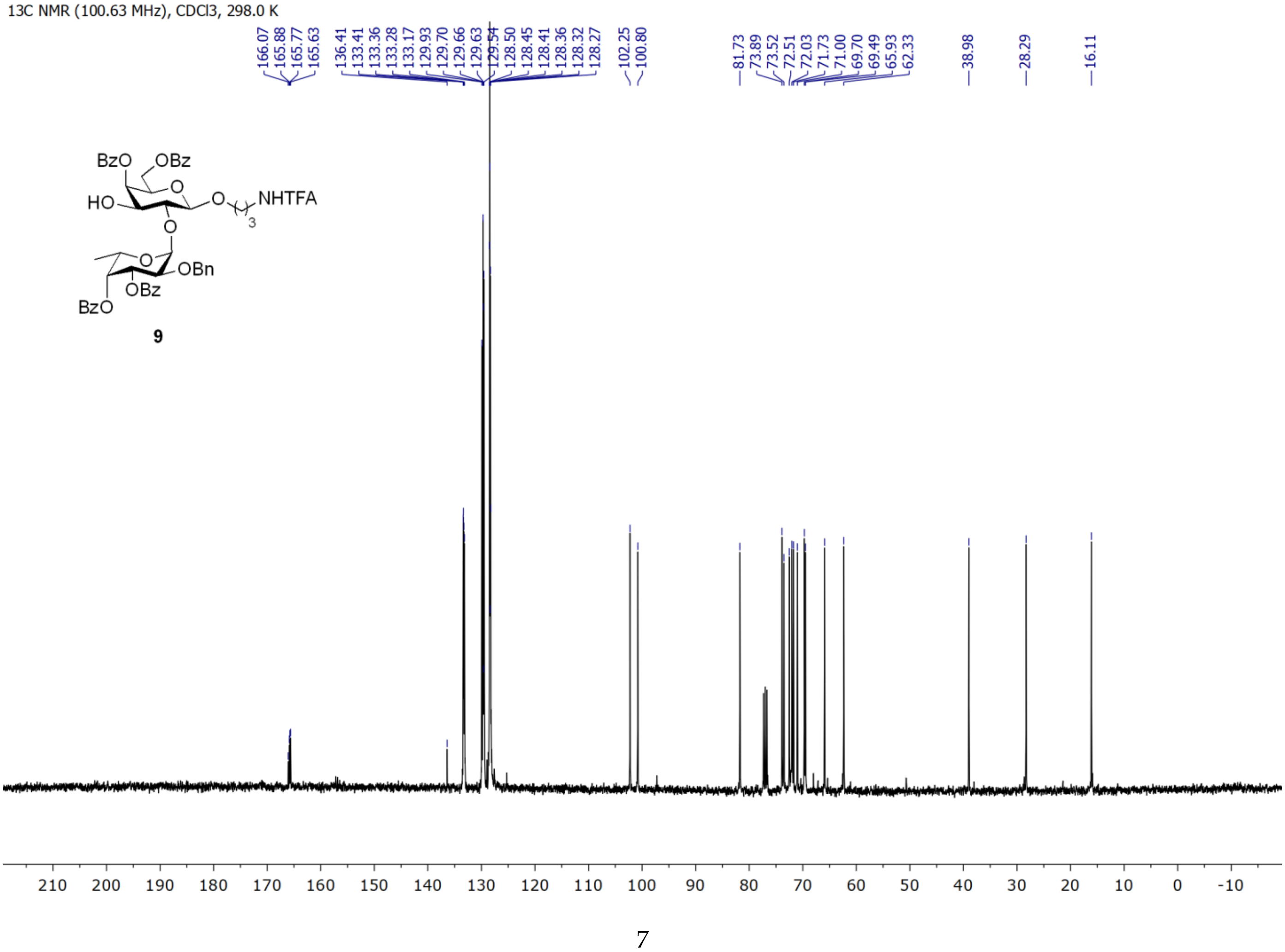

^{1}H NMR of compound **13**

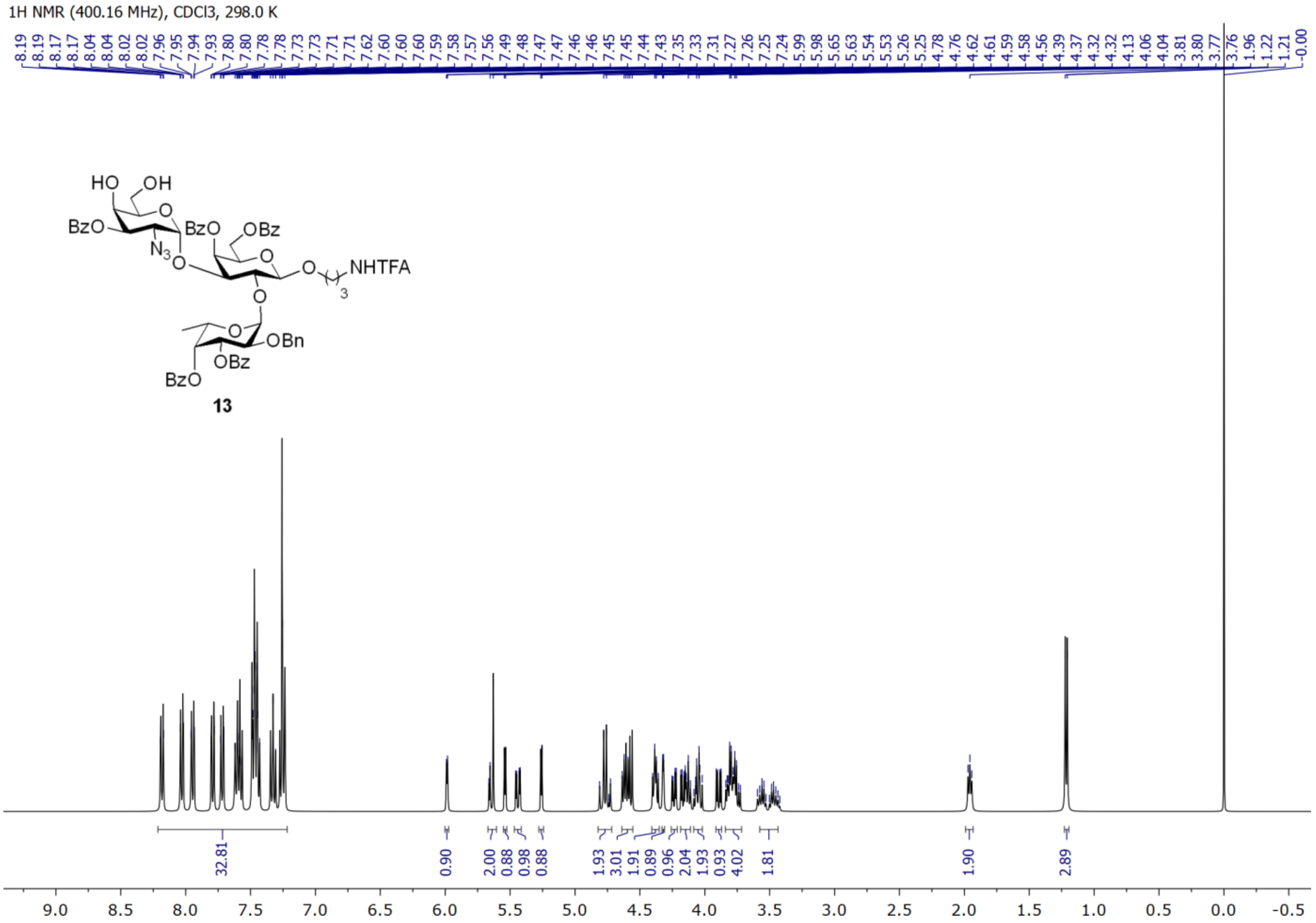

^{13}C NMR of compound **13**

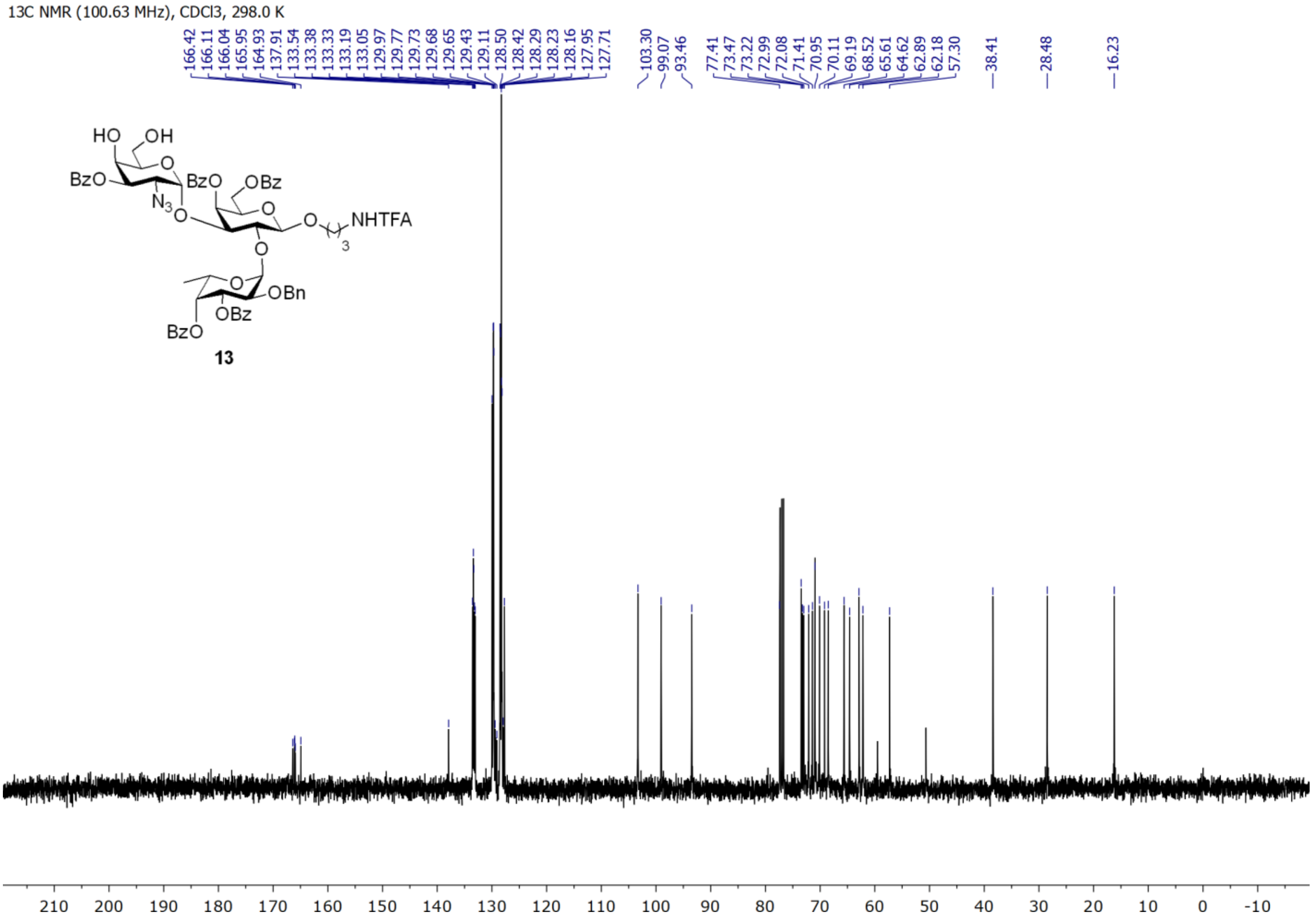

^{1}H NMR of compound **14**

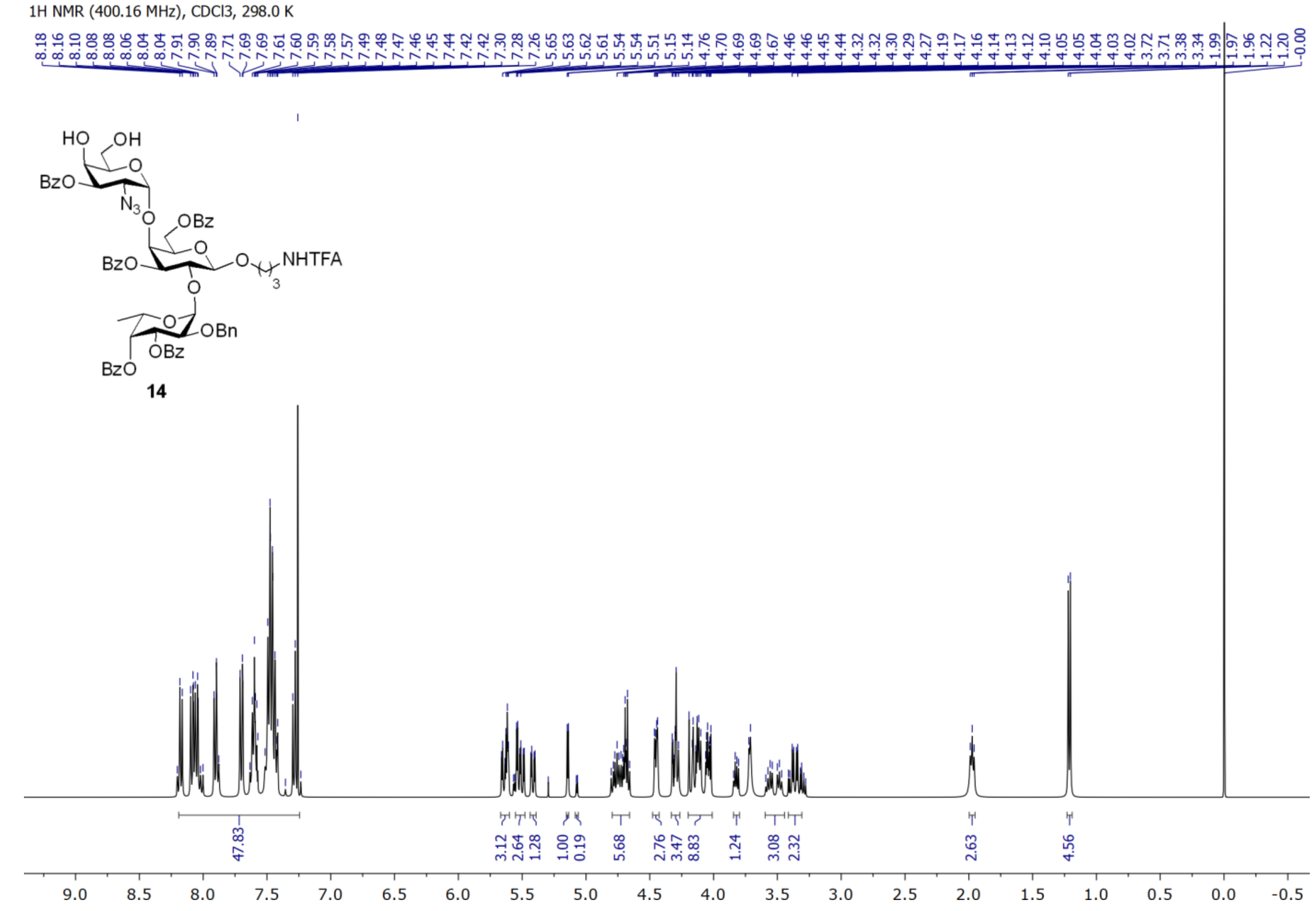

^{13}C NMR of compound **14**

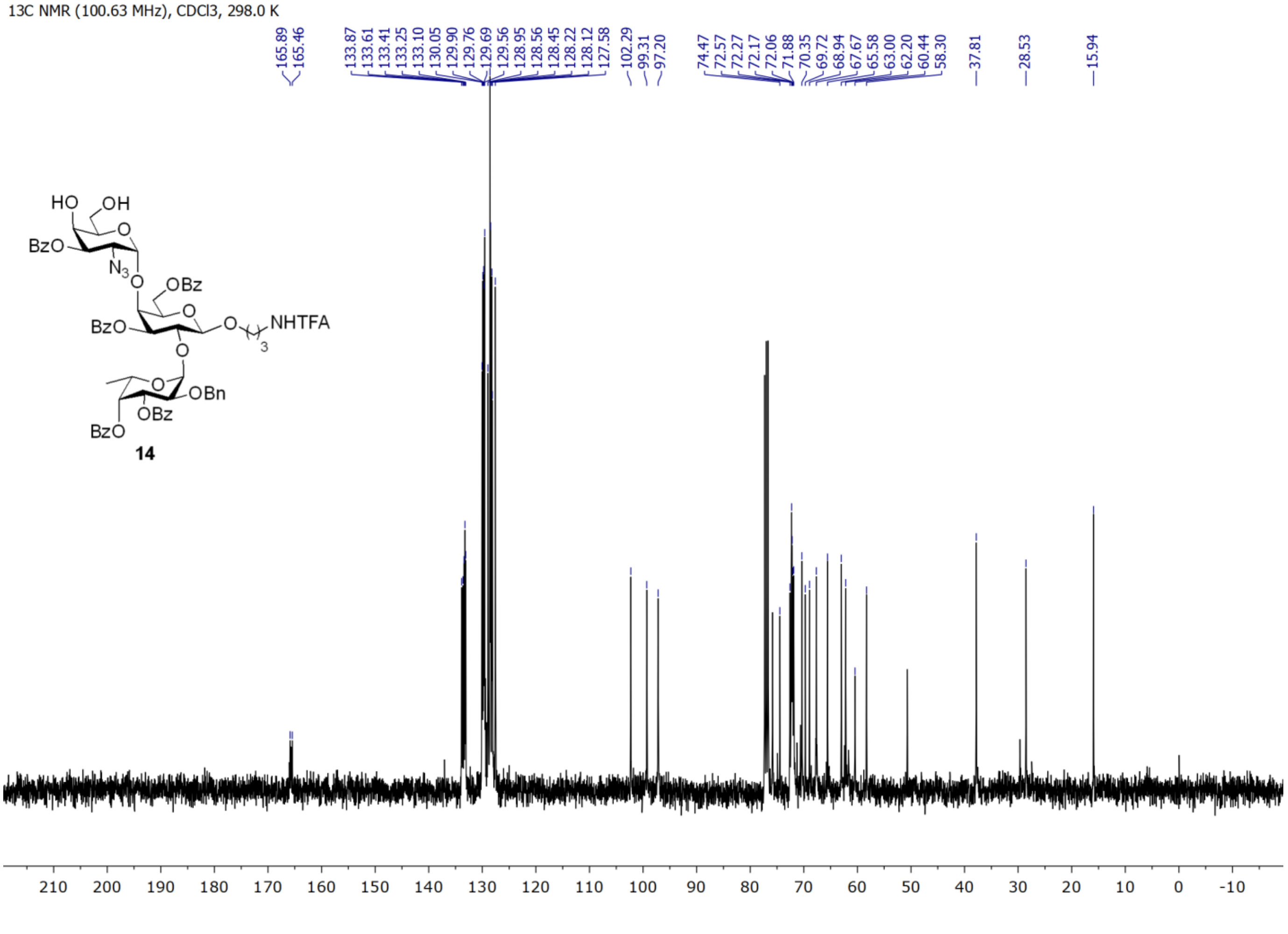

Supplement: Supplementary file 1 [file molecules-26-05887-s001.zip › molecules-1376410-supplementary.pdf]
